# Breastfeeding Knowledge and Behavior Among Women Visiting a Tertiary Care Center in India: A Cross-Sectional Survey

**DOI:** 10.5334/aogh.2093

**Published:** 2019-05-03

**Authors:** Priya Sultania, Nisha R. Agrawal, Anjali Rani, Dinesh Dharel, Rachael Charles, Rajesh Dudani

**Affiliations:** 1 Institute of Medical Sciences, BHU, Department of Obstetrics and Gynecology, IN; 2 University of Calgary Cumming School of Medicine, Division of Neonatology, Department of Pediatrics, CA; 3 University of Arizona College of Medicine, Division of Neonatology, Department of Pediatrics, US

## Abstract

**Background:**

Breastfeeding is commonly practiced by a majority of mothers in developing countries, though there are widespread misconceptions about optimal breastfeeding traditions. In addition to culturally prominent rituals and social norms, incorrect and inadequate breastfeeding knowledge is major factors for this high-risk behavior.

**Objectives:**

To assess knowledge, attitude and practices of breastfeeding among girls and women visiting a tertiary care center in India and to find out the factors, which influence the breastfeeding behaviors.

**Design/Methods:**

It is a cross-sectional, questionnaire-based study done among women attending outpatient and inpatient Department of Obstetrics & Gynecology of S.S. Hospital, Banaras Hindu University, India. A face-to-face interview using a pre-designed, self-administered, standardized questionnaire regarding knowledge, attitude, and practices of breastfeeding was conducted. The information was collected and analyzed using SPSS statistical software.

**Findings:**

Among 1000 women enrolled in the study, 89% were married, 25% were primiparous, and 52% were multiparous. More than 50% were illiterate, 91% unemployed, and 90% had hospital delivery. Of the total 770 mothers, only 55% received proper antenatal care during pregnancy, of which only 40% were counseled about breastfeeding. Regarding knowledge and attitude about breastfeeding, majority females (71.4%) considered breast milk as best food for a newborn, which was better in younger women <20 years (86%). Regarding breastfeeding behavior, only 45% mothers initiated breastfeeding within one hour of delivery, which was worse in home delivered mothers (25%). Most (82%) mothers fed colostrum to their babies but 27% of mothers gave pre-lacteal feeds. Illiterate mothers (56.3%), mothers with only primary education (70%), and unemployed mothers (53.85%) continued to do exclusive breastfeeding without initiating complementary feeds even after six months.

**Conclusion(s):**

Although breastfeeding is practiced by a majority of mothers in a developing country like India, there is a significant gap in knowledge and optimal breastfeeding behaviors. Healthy breastfeeding behavior can be encouraged among mothers by proper counseling by health care workers and organizing educational programs focusing women especially with low education and limited resources.

## Background

Breastfeeding is commonly practiced by a majority of mothers in developing countries, though there are widespread misconceptions about optimal breastfeeding traditions. Breast milk is the first natural food for a baby that provides all the energy and nutrients that the infant needs for the first few months of life. Breastfeeding has both short-term and major long-term effects on the health, nutrition, and development of the child and mother’s health. Appropriate breastfeeding practices prevent child mortality and morbidity from diarrhea, respiratory and other infections, otitis media, necrotizing enterocolitis, and sudden infant death syndrome [1]. The maternal benefit of breastfeeding in birth spacing by promoting lactational amenorrhea and reduction in breast and ovarian cancer has been well established [1].

Optimal breastfeeding practices include exclusive breastfeeding for the first six months of life, early initiation of breastfeeding within one hour of life, and continued breastfeeding for up to and beyond two years of age [2]. World Health Organization (WHO) also recommends providing colostrum from the beginning and timely initiation of nutritionally adequate, safe, age-appropriate complementary feeding at six months of life. Nearly half of all deaths in children under five years of age are attributable to under-nutrition [3]. Optimal breastfeeding of infants under two years of age has the greatest potential impact on child survival of all preventive interventions, with the potential to prevent about 13% of all deaths in children under five in the developing world [4]. Although more than 80% of neonates receive breast milk in nearly all countries, only about half begin breastfeeding within the first hour of life, and rates of exclusive breastfeeding are well below 50% in most of the countries [5].

Not breastfeeding or suboptimal breastfeeding is associated with lower intelligence and economic losses of about $302 billion annually or 0.49% of the world gross national income [6].

India has the highest number of child deaths in the world and accounts for 20% of the 5.9 million global child deaths [7]. India under five mortality and infant mortality rate are 48 and 38 per 1000 live births, respectively (UNICEF India Statistics 2015) and almost 50% of it is attributable to malnutrition [8]. Exclusive breastfeeding for six months of age is 65% and early initiation of breastfeeding within one hour is 45% (UNICEF India statistics 2015). Both of these fall far below the recommended practice.

Optimal breastfeeding practices are potentially one of the top interventions for reducing under-five mortality and is essential for the achievement of many of the newly launched Sustainable Development Goals by 2030, as it can help to improve child and maternal health, nutrition, economy, intelligence, and human capital, while reducing inequalities.

There are several determinants for optimal breastfeeding practices, which include socio-cultural factors, factors associated with health system and services, family and community, workplace and employment, mother-infant relationship, and mother and infant attributes. It has been shown by several studies that if relevant interventions are delivered adequately using supportive measures to address these determinants, breastfeeding practices can improve rapidly.

This study assessed knowledge, attitude, and practices of breast feeding among women and the factors which influence breastfeeding in an attempt to facilitate the appropriate promotion and health support of breastfeeding mothers and to contribute to the overall increase of the breastfeeding rate. Information about breastfeeding practices in the suburban/rural population will be useful for policy makers and for interventional programs.

## Methods

### Study Setting

Women attending outpatient and inpatient Department of Obstetrics and Gynecology, S.S. Hospital, Banaras Hindu University were included in this study.

Study Period: June 2014 to June 2016.

Sample Size: 1000.

Sample size was calculated using the formula: N = 4pq/r^2^.

Where p is the prevalence of exclusive breastfeeding for six months in India, per Demographic Health Survey 2016, which was 65%, q = 1 ’ p and r is taken as 5% margin of error for p.

Using this formula, the sample size will be 862, and oversampling of 15% for noncompliance will make approximately 1000.

### Study Design

It is a cross-sectional, questionnaire-based study done in the setting of a teaching hospital predominantly catering to the health needs of the population of Varanasi, India.

### Method of Data Collection

All women visiting the Obstetrics and Gynecology outpatient and inpatient Department of S.S. Hospital, Banaras Hindu University were approached for participation in the study. After obtaining the informed consent, a face-to-face interview using a pre-designed, self-administered, standardized questionnaire regarding knowledge, attitude, and practices of breastfeeding was conducted.

The questionnaire, included data about maternal age, parity, type of delivery, place of delivery, education, employment, socio-economic status, religion, residence initiation, and duration of exclusive breastfeeding and weaning practices.

### Inclusion Criteria

All girls and women visiting the Obstetrics and Gynecology outpatient and inpatient Department of S.S. Hospital, Banaras Hindu University.

### Exclusion Criteria

Conditions where breastfeeding is contraindicated like galactosemia, mother suffering from cancer, active tuberculosis, and psychoses.

### Statistical Analysis

The information was collected and analyzed using SPSS statistical software (Version 16.0). Descriptive statistics such as mean, frequency, and percentages of various parameters were calculated. Chi-Square Test and Fischer’s Exact Test were used to deduce the association between knowledge, attitude, and practices of breastfeeding with different attributes. The p value < 0.05 was considered statistically significant.

## Results

The age of the participants in this study ranged between 15–60 years with maximum belonging to the 20–30-year age group. Of the participants, 89% were married. Unmarried girls were also included in this study to gather information about knowledge regarding breastfeeding in the younger generation. Most people were Hindu by religion. Most participants (54%) did not have any formal education. Only 3% were graduate and above. This is probably because we catered to an Indian state population where illiteracy is highest, i.e. UP and Bihar. Of the women, 49% belonged to low socio-economic background; only 3% belonged to rich class according to Modified BG Prasad classification. The majority (91%) of the participants in our study were unemployed (Table 1).

**Table 1:** Socio-demographic characteristics: (N = 1000).

Factors	Respondents (%)	Factors	Respondents (%)

Age		Parity	
<20 years	130 (13%)	Unmarried	110 (11%)
20–30 years	546 (55%)	Nulliparous	120 (12%)
30–40 years	206 (20%)	Primipara	251 (25%)
>40 years	118 (12%)	Multipara	519 (52%)
Marital status		Employment	
Unmarried	110 (11%)	Yes	90 (9%)
Married	890 (89%)	No	910 (91%)
Religion		Nutritional Status (BMI)	
Hindu	738 (74%)	<18.5	238 (24%)
Muslim	244 (24%)	18.5–24.9	620 (62%)
Others	18 (2%)	>25	142 (14%)
Education		Socioeconomic status	
No formal education	538 (54%)	Class I	28 (3%)
Primary education	186 (18%)	Class II	49 (5%)
High school	130 (13%)	Class III	112 (11%)
Intermediate	120 (12%)	Class IV	318 (32%)
Graduate and above	26 (3%)	Class V	493 (49%)

From the table 2 it is evident that the majority (71.4%) of women had general knowledge about breastfeeding and that breast milk is the best milk. Of the women, 64% knew that the child remains healthy and 46% knew breast milk to be more nutritious. Very few mothers knew about fore milk & hind milk.

**Table 2:** Women knowledge and attitude towards breastfeeding (Multiple responses).

Knowledge	Respondents (%)	Attitude	Respondents (%)

Child remains healthy	636 (64%)	Breast feeding leads to loss of figure	5 (0.5%)
More nutritious	460 (46%)	Breast feeding is old fashioned	21 (2%)
Gives natural immunity	116 (12%)	Is pure & costs nothing	519 (52%)
Helps in preventing conception	138 (14%)	Fosters close bond	710 (71%)
Mothers milk is the best milk	714 (71.4%)	In public, it is embarrassing	220 (22%)
Improves growth & development	92 (9%)	Prevents going to work	56 (6%)
Fore milk & hind milk	2 (0.2%)		

The majority of mothers had a favorable attitude towards breastfeeding. Of the women, 71% thought that breast milk promotes bonding between mother and child and 52% thought it is pure and cost effective (Table 2). Very few mothers felt that breastfeeding is old-fashioned, embarrassing in public, and leads to loss of figure.

It was noticed only 45% of mothers initiated breastfeeding during the first hour of birth and 27% of mothers gave pre-lacteal feeds (Table 3). In this study colostrum was given by 82% of mothers. Out of 770 cases, 47% of mothers exclusively breastfed for six months and less while 53% breastfed for >6 months. One third of mothers used a bottle to feed the infant. While 91% of our mothers breastfed on demand, 78% of them only fed once or twice during the night and 74% of mothers did not use pacifiers.

**Table 3:** Practices regarding breastfeeding (n = 770) [excluding unmarried & nullipara].

Practices	Respondents (%)	Practices	Respondents (%)

Initiation of Breastfeeding		Complementary feed initiation	
<1 hour	346 (45%)	<1 month	94 (12%)
1–6 hours	270 (35%)	1–3 months	102 (13%)
6–24 hours	56 (7.3%)	3–6 months	182 (24%)
>1 day	98 (12.7%)	>6 months	392 (51%)
Duration of exclusive Breast feeding		Pre- lacteal feed	
<1 month	128 (16.6%)	Given	208 (27%)
1–6 months	234 (30.4%)	Not given	562 (73%)
>6 months	408 (53%)		
Bottle feed		Colostrum Feeding	
Given	250 (33%)	Fed	631 (82%)
Not given	520 (67%)	Not Fed	139 (18%)

Regarding breastfeeding technique, most of the mothers (85%) fed mostly in sitting down position and 92% of them practiced burping after feeds. Of the mothers, 84% were placing the nipple and most of the areola inside the child’s mouth and completely emptying one breast followed by the other. Most mothers were practicing correct breastfeeding technique but only 54% mothers practiced hand washing before feeds.

A mother’s perception of breast milk being inadequate (41%) was the most common reason for stopping breastfeeding, followed by introduction of bottle milk (30%). Only 0.6% mothers cited an inverted nipple, cracked nipple or breast abscess as reasons for stopping breastfeeding (Table 4).

**Table 4:** Reasons for stopping breastfeeding (multiple responses).

Reasons	Respondents (%)	Reasons	Respondents (%)

Working mother	28 (4%)	Subsequent pregnancy	5 (0.6%)
Mother felt that breast milk was inadequate	320 (41%)	Inverted nipple, cracked nipple, or breast abscess	4 (0.6%)
Infant taking top feeds	120 (15%)	Embarrassment in breastfeeding	17 (2%)
Introduction of bottle milk	230 (30%)	Mother felt that duration of breastfeed was adequate	150 (19%)

In our study only 55% women had regular antenatal checkups and only 40% received antenatal counseling regarding breastfeeding. Of the deliveries, 63% were normal, spontaneous vaginal delivery; 34% delivered by LSCS, and 3% by instrumental deliveries. As the interview was conducted in tertiary care hospital, 90% of our cases were delivered in the hospital.

Regarding the source of information for breastfeeding, 61% received information from family/friends, 57% from past experience, 39% from media/literature, and only 35% from health professionals (Table 5).

**Table 5:** Source of information regarding breast feeding (multiple responses).

Sources	Respondents (%)	Sources	Respondents (%)

Health personnel	345 (35%)	Media or literature	386 (39%)
Family/friend	613 (61%)	Previous experience	510 (57%)

From the above data, it can be concluded that mothers with no or lower education, unemployment, and lower socioeconomic status were exclusively breastfeeding for longer duration and it was statistically significant.

From the above data, it can be seen that a higher number of hospitals delivered infants and infants delivered by normal vaginal delivery were initiated breastfeeding within one hour of life which is statistically significant.

## Discussion

### Knowledge and Attitude of Breast Feeding

This study was done in the setting of medical college hospital predominantly catering the need of suburban/rural population of eastern Uttar Pradesh and Bihar. The findings reveal that majority of mothers have good knowledge about breastfeeding and believe breast milk as the best milk (71.4%), more nutritious (46%), and cost efficient (52%). In addition, 71% women thought that breast milk promotes bonding between mother and child, which is similar to a study done by Eman S Mohammed et al. (2014), in Egypt, that showed 76.8% [9]. Only 14% of mothers had knowledge about its function as lactational amenorrhoea, i.e. role of breastfeeding in contraception, which is quite dismal for a developing country like India where contraception plays a big impact on population control. A study in West Bengal by Abhay C. Pal et al. (2014) showed that 68% of mothers had knowledge regarding contraceptive effect of breast milk, which is much higher in comparison to our study, but most of the other studies in India have shown similar results as ours [10,11]. This could be due to different education level of the participants. There was no significant association in knowledge of breastfeeding with regard to various demographic variables such as age, religion, or marital status in our study.

In this study only 310 (40%) mothers received antenatal counseling regarding benefits and management of breastfeeding. This number seems inadequate and demands that emphasis should be laid on promoting breastfeeding starting from antenatal period. The majority (61%) came to know about breastfeeding prior to conception from family and friends, which is a positive trend and this emphasizes the importance of educating the whole society regarding the benefits of breastfeeding and its proper practice.

### Practices of Breast Feeding

There is quite a gap between knowledge and practice between breastfeeding. Edmond et al., in Ghana, showed that 22% of infants would be saved from death if all women initiated breastfeeding within one hour of birth [12]. In this study 45% of mothers initiated breastfeeding within one hour of delivery. The early initiation of breastfeeding within one hour of birth is 41.6% nationally, which is similar to our study (National Family Health Survey 4 NFHS 4 2015–2016). This rate varies from 27.8% (Uttarakhand) to 73.3% (Goa) in different states of India (NFHS 4). In some states like Bihar it has increased from 3.7% to 34.9% between National Family Health Survey 3 and National Family Health Survey 4. In a study by Petro S et al. (2012), in tribal people in Orissa it was as low as 8%. This rate has increased significantly in most of the states in India due to increased facility-based delivery and tremendous effort placed to improve nutritional indicators by government and international agencies in the last decade. There was significant correlation between type of delivery and early initiation of breastfeeding in our study, with nearly double the number of mothers with normal vaginal delivery who breastfed their baby within one hour compared to those undergoing a caesarean section. However, at the end of six hours about 70% of mothers initiated breastfeeding in both groups. Similar phenomenon was observed in terms of place of delivery, where hospital -delivered babies were breastfed earlier than those born at home (p < 0.001, Table 7).

Some of the main reasons for the delay are lack of knowledge, inadequate lactation, shifting of baby to nursery for observation in case of hospital delivery, less efforts from hospital staff, and postoperative pain in mothers with LSCS.

Pre-lacteal feeds were still being given in about 27% cases, which should be discouraged as it could have a bearing on neonatal morbidity and mortality. In a study in Madhya Pradesh, India by Tiwari et al. (2007), the majority of families had the habit of pre-lacteal feeding; 43% gave honey as the first feed followed by cow’s milk (13.2%), or plain water in 13.2% cases [13]. In our study honey, plain water, and glucose water were most common pre-lacteal feeds and honey was used by majority of mothers (65%) who gave pre-lacteal feeds.

There is a belief in women that the child swallows waste and impurities in the womb and pre-lacteal feeds were given to cleanse their system [14].

Colostrum was being fed in 82% of cases in this study, which was similar to that in Madhya Pradesh where it was 90% [13]. However, a study from rural Karnataka India by Benakappa et al. (1989), showed that colostrum was rejected by 58% [15], but a similar study in villages of central Karnataka by Banapurmath et al. (1996), showed much improved rate where 71.4% of mothers fed colostrum to their babies. The rate is also low (53%) in African countries such as Nigeria [16].

The main reason for not feeding colostrum in our study was the belief that thick yellowish colostrum is harmful and difficult to digest by the newborn. There was no correlation between colostrum feeding and religion. The majority of Hindus and Muslims believed colostrum to be healthy for the baby.

In our study, 47% of mothers exclusively breastfed for six months or less and 53% exclusively breastfed for more than six months, which was worse than the national average rate of 65%. While comparing the duration of breastfeeding mothers with lower education (literate 56%, primary 70%), unemployed (53.9%), and mothers with poor socioeconomic status class 4 (54.1%) and class 5 (57.3%) continued to breastfeed for a longer duration (Table 6). Tewabe et al. (2015), Ethiopia, showed similar correlation of exclusive breastfeeding with low-income mothers and unemployed mothers [17]. High prevalence of malnutrition in children in rural areas may be related to prolonged exclusive breastfeeding. Breast milk alone is not sufficient to fulfill the nutritional needs to sustain optimal growth beyond six months. Therefore, mothers should be counseled properly about starting of complementary feeds.

**Table 6:** Correlation of duration of exclusive breastfeeding with education and socioeconomic factors.

Factors	Duration		

Education (p-value = < 0.001)	<1month	1–6 months	>6 months

No formal education	70 (17.2%)	108 (26.5%)	230 (56.3%)
Primary	22 (15%)	22 (15%)	103 (70%)
High school	17 (16.1%)	47 (44.3%)	42 (39.6%)
Intermediate	18 (21.1%)	42 (49.4%)	25 (29.4%)
Graduate and above	1 (4.2%)	18 (75%)	5 (20.8%)
**Employment (p-value = 0.005)**			
Yes	4 (9.5%)	22 (52.4%)	16 (38%)
No	124 (17%)	212 (29.1%)	392 (53.9%)
**Socioeconomic status (p-value = 0.0004)**			
Class 1	3 (15.8%)	10 (52.6%)	6 (31.6%)
Class 2	5 (13.2%)	22 (57.9%)	11 (28.9%)
Class 3	9 (11.7%)	33 (42.8%)	35 (45.5%)
Class 4	51 (19.8%)	67 (26.1%)	139 (54.1%)
Class 5	60 (15.8%)	107 (26.9%)	212 (57.3%)

**Table 7:** Correlation between initiation of breast feed with mode of delivery.

Factors	Time of Initiation of Breastfeeding			

Place of Delivery (p-value < 0.001)	<1 hour	1–6 hours	6–24 hours	>24 hours

Home	20 (25.3%)	42 (53.1%)	13 (16.5%)	4 (5.1%)
Hospital	326 (47.2%)	228 (33.1%)	43 (6.2%)	94 (13.6%)
**Mode of Delivery (p-value < 0.001)**				
Normal vaginal	188 (38.7%)	143 (29.4%)	99 (20.4%)	56 (11.5%)
LSCS	53 (20.5%)	114 (44.2%)	49 (19%)	42 (16.3%)
Operative vaginal	5 (19.2%)	13 (50%)	8 (30.8%)	0 (0%)

Several studies have shown that the mean duration of exclusive breastfeeding in India ranges from 6.7 months in Tamil Nadu to 10.8 months in Andhra Pradesh [18]. In a study by Maheswari et al. (2012), in Pondicherry, only 38% of the mothers knew that exclusive breastfeeding should be given for six months [18]. Delayed weaning is one of the major causes of malnutrition among infants in developing countries [19]. Ineffective communications from health care professionals, inadequate knowledge, poverty, and unemployment are the main reasons for this practice.

In the present study bottle-feeding was given by 33% of mothers. The main reason for starting bottle-feeds was that most mothers felt that breast milk was inadequate. Most mothers started bottle-feeding before six months. There is an urgent need to educate the mothers regarding disadvantages of bottle-feeding. This practice should be eliminated completely in developing countries like India where infection rate in neonates and infant is significantly high. A study by Patel, et al. (2015), showed bottle feeding rate of 18.4% whereas study by Das et al. (2013) showed bottle feeding rate of 28.1% [20,21]. A similar study in two rural districts of Sindh, Pakistan showed the bottle-feeding rate at 12% [22]. It was 76% in a study shown by Shamim et al. [23]. The bottle-feeding rate varies significantly in different studies and the higher rate could be attributed to illiteracy, mothers’ old age, and increased parity [23]. The higher rate in our study could be due to urbanization and poor literacy rate among our mothers.

Also, in the present study, complementary feed was started before six months by 49% of mothers. Furthermore, introduction of complementary feeding was started by 12% before one month of age; by 13% at 1–3 months of age; by 24% at 3–6 months of age, and by 51% after six months of age. Early introduction of complementary feeding has the disadvantage on breast milk production and can affect families of low socio-economic status in terms of cost of food. The mean age of starting complementary feeding in this study was around six months, which is in accordance with WHO recommendation. In this study breastfeeding continued along with complementary feeding which is a good practice.

In our study the main reason (41%) for stopping breast-feeds was the thought breast milk is insufficient for the child. A study by Pal et al. (2014), in West Bengal, showed similar results where 42% of the mothers’ concern was that their milk is insufficient for the baby. In several other studies also, insufficient breast milk was given as the most common reason for stopping breastfeeding [24,25,26].

Maternal perception about inadequacy of breast milk is the most likely reason for shifting to formula feeding while introducing bottle-feeds in less than six months. Another reason for the high rate of using ready-made liquid formula in the hospital could be poor communication and advocacy from health professionals.

In the present study, breastfeeding technique was good in most of the mothers: 85% fed in sitting position, 84% knew to keep nipple and areola inside mouth, and 54% knew about cleanliness of breast and hand washing before feeds. Of the mothers, 92% knew about burping after feeds and 84% knew about emptying of one breast followed by the other. Therefore, breast-feeding technique was appropriate in most mothers. A study by Benakappa et al. (1989), in rural Karnataka, showed that 42% mothers did not practice burping after feeds. But a study in a tertiary care center in Karnataka by Vijayalakshmi et al. (2015), showed most of the mothers (91.8%) were aware of the importance of burping after feed which was similar to our study. In a study by Premlata et al. (2014), in Rajasthan, it showed only 35% mothers knew about proper position but 78.2% mothers practiced burping. These findings from the present study clearly highlight the fact that adequate breastfeeding counseling is lacking during antenatal, intra-partum, and postpartum period. Counseling should focus on mainly educating women on their practices of breastfeeding.

## Conclusion

Breastfeeding is practiced by a majority of mothers in a developing country such as India, with high rate of continued breastfeeding beyond six months. However, there is a significant gap in knowledge and optimal breastfeeding behaviors among mothers. Healthy breastfeeding behavior can be encouraged among mothers by receiving proper counseling conducted by health care workers and organizing educational programs focusing on women especially with low education and limited resources. We would like to highlight following recommendations which may help to promote successful breastfeeding behavior:

- All health care workers who care for women and young children should be trained on breast feeding counseling.
- These health care workers include: midwives, community health workers/traditional birth attendants, pediatric/obstetric nurses and doctors who usually work in maternity/pediatric facilities, hospitals, community health centers, and health posts.
- Policy should be placed to assess breastfeeding behavior at each visit, during the pregnancy and postnatal periods.
- Special workshops should be arranged during these periods for women and family members to disseminate knowledge and skills needed for proper breast feeding.
- Health care workers should be able to: communicate the benefits of breastfeeding for both mother and baby, demonstrate the proper techniques to breastfeed a baby, and assess actual and potential difficulties/barriers and help the women overcome them.
- During counseling, heath care workers should discuss: the importance of exclusive breast feeding for first six months, the benefits of initiating skin-to-skin contact as soon as possible following the birth (to facilitate early initiation of breastfeeding), and continued breastfeeding along with appropriate complementary foods up to and beyond two years of age.
- Special support groups or organizations working in the community, who may support women who are breast feeding, should be identified and involved.
- In order to have a successful promotion and fostering of optimal breastfeeding practices in the community, it is important to have an alliance and collaboration of multi-stake holders, including: local government/non-profit organizations, health care systems, community, local support groups, and family members.

## Date Accessibility Statement

The datasets used and/or analyzed during the current study are available from the corresponding author on reasonable request.

## Abbreviations

World Health Organization (WHO), National Family Health Survey (NFHS)
